# High Expression of Polo-Like Kinase 1 Is Associated with Early Development of Hepatocellular Carcinoma

**DOI:** 10.1155/2014/312130

**Published:** 2014-06-11

**Authors:** Wei Sun, Qi Su, Xiankui Cao, Bin Shang, Aishan Chen, Hongzhuan Yin, Baolin Liu

**Affiliations:** Department of Surgery, Shengjing Hospital, China Medical University, Sanhao Road, Shenyang 11004, China

## Abstract

Polo-like kinase 1 (PLK1), one of serine/threonine-protein kinase, has been demonstrated to play pivotal roles in malignant transformation. Here we illustrated the clinicopathological significance of PLK1 expression in hepatocellular carcinoma (HCC) in more detail. Immunohistochemistry was performed to detect the expression of PLK1 in 67 HCC patients as well as corresponding noncancerous liver tissues. In addition, the correlation of PLK1 expression with clinicopathological factors or prognosis of HCC was analyzed. Results showed that the expression of PLK1 was increased significantly in HCC tissues than that of corresponding normal liver tissues. The correlation between PLK1 and HCC cell differentiation or capsule invasion was also revealed. We found that PLK1 inhibition promoted cell arrest in G2/M phase of cell cycle and cell apoptosis. Our results also indicated that the potential mechanisms of PLK1 inhibition regulating cell growth involved enhancing expression of caspase3, caspase8, and Bax and decreasing expression of Bcl-2. Furthermore, we also found that PLK1 downregulation inducing inhibition of cell growth was associated with enhancing expression of p53. Thus, we presume that the status of PLK1 expression might be an independent prognostic factor for HCC and targeting PLK1 might be a useful strategy for diagnosis and treatment of human HCC.

## 1. Introduction

Cancer is a complicated disease that develops slowly due to gradual accumulation of genetic and epigenetic alterations over time [1, 2]. Tumor cells often harbor mutations followed by activated oncogene expression and inactivated tumor-suppressor expression. These alterations combined with dysregulation of cell division, one of the hallmarks of the cancer phenotype [1]. In spite of the universal property of tumors, many important issues remain to be illustrated, including whether the order of the unsuccessive alterations is critical to cellular malformation and how mutations of cancer pathways are involved in the development of cancer disease.

Polo-like kinases (PLKs) as a family of proteins that has a significant role in the maintenance of mitotic integrity have attracted attention. Currently, five members of the polo-like kinase family have been identified in human (PLK1-5) [3]. Among them, PLK1 is best studied which is essential mitotic kinase that controls mitotic entry, centrosome maturation, bipolar spindle formation, cohesion dissociation, and chromosome congression and segregation, as well as cytokinesis [4–6]. The levels of PLK1 rise in G2 phase and arrive at the peak in M phase. The expression of PLK1 is increased in many types of tumor, including cancer in brain, breast, colon, head and neck, lung, pancreas, bile duct, bladder, and prostate as well as hepatocellular carcinoma (HCC). Moreover, its expression often correlates with poor diagnosis [7]. Its overexpression results in multinucleation and overrides the G2 arrest checkpoint which is induced by DNA damage. Meanwhile, the p53 tumor-suppressor protein is phosphorylated by PLK1, which can inhibit the proapoptotic function of p53. The inhibition of PLK1 leads to a failure to complete mitosis, eventually resulting in cell death [8]. Therefore, PLK1 expression shows a close relationship with cancer development and its small molecule inhibitors have been used in clinical trials in advanced cancer patients.

HCC is the third most common cancer in the world with more than 500,000 deaths a year [9]. It is reported that the number of HCC cases increases every year [10]. Although several factors for HCC have been revealed, including infection with hepatitis B virus (HBV) and hepatitis C virus (HCV), there is still no effective treatment for this cancer type at present, for the potential mechanisms in both molecular level and cellular level of HCC pathogenesis remained poorly clarified [11–13]. Previous studies have revealed that centrosome abnormality and upregulation of PLK1 were observed in HCC [14, 15]. So far, the correlation of PLK1 and HCC in clinicopathological variable is not well described and the corresponding potential mechanism should be illustrated.

In this study, we demonstrated that PLK1 was expressed in human multiple HCC cell lines (HepG2 and BCL-7402) and liver samples from HCC patients. We found that increased PLK1 expression not only is an adverse prognostic factor for HCC but also is associated with HCC early development. Our further studies demonstrated that PLK1 inhibition by siRNA can significantly downregulate hepatocellular cell cycle progression and promote cell apoptosis. Therefore, we presume that PLK1 expression can be the important marker for HCC diagnosis; moreover, PLK1 may be the target for cancer treatment.

## 2. Methods

### 2.1. Human Tissue Samples

Normal livers (*n* = 5), 67 HCCs patients (*n* = 67, which including 27 cases). Normal livers were from autopsy cases of healthy individuals. The clinical data of patients involved in this study was shown in Table 1. Liver tissues were kindly provided by Department of Pathology, Shengjing Hospital, China Medical University. The protocol of this study was approved by the ethics committee of the institution involved, and informed consent was obtained from all the subjects.

### 2.2. Cell Line and Treatment

HepG2 and BCL-7402, two human HCC cell lines, were provided by Department of Cell Biology, Institute of Basic Medical Science, China Medical University. The sequences of small interfering RNA (siRNA) were as follows:

 siRNA1: sense: 5′-UGAAGAAGAUCACCCUCCUdTdT-3′;
 antisense: 5′-AGGAGGGUGAUCUUCUUCAdTdT-3′;
 siRNA2: sense: 5′-GCACCGAAACCGAGUUAUUdTdT-3′;
 antisense: 5′-AATAACTCGGTTTCGGTGCdTdT-3′;
 siRNA negative control: sense: 5′-UUCUCCGAACGUGUCACGUTT-3′;
 antisense: 5′-ACGUGACACGUUCGGAGAATT-3′.

### 2.3. Cell Apoptosis and Cell Cycle Analysis

For apoptosis assay, apoptotic cells were measured by staining with annexin V-PI provided in an apoptosis detection kit (BD Pharmingen, USA), according to the manufacturer's instructions. Stained cells were analyzed with a Flow cytometry. Analysis of cell cycle distribution was performed by means of flow cytometry of 2,4-diamidino-2-phenylindole-stained nuclei21 with BD FACS calibur using the multicycle program.

### 2.4. Western Blot Analysis

For western blot testing, cells were lysed with RIPA (150 mM NaCl, 10 mM Tris-HCl [pH 8.0]), 1% Nonidet P-40, 0.5% deoxycholic acid, 0.1% sodium dodecyl sulfate [SDS], and 5 mM EDTA containing 0.7% phenylmethylsulfonyl fluoride and proteinase inhibitor mixture. Protein aliquots (30 *μ*g) for detection of PLK1 and p53 were mixed with an equal amount of 2 × SDS sample buffer, boiled at 98°C for 5 min, centrifuged, and separated by 10% SDS-PAGE. The gels were then transferred onto polyvinylidene fluoride membranes (Millipore, MA). Membranes were blocked with 5% nonfat milk powder in Tris-buffered saline (TBS)/Tween 20 (TBS/T) and washed three times with TBS/T. Primary anti-p53 antibody (Santa Cruz Biochemicals, USA), anti-PLK1 antibody (Merck Drugs & Biotechnology, USA) were added in TBS/T containing 5% bovine serum albumin at suitable dilution and kept overnight at 4°C. Membranes were washed with TBS/T, and horseradish peroxidase- (HRP-) conjugated secondary IgG (Cell Signaling Technology, USA) were added at a dilution of 1/2,000 in TBS/T for 1 h at room temperature (RT). For control of equal protein loading, membranes were incubated overnight with primary antibody to *β*-actin (Sigma, USA) (1/1,000; diluted in TBS/T containing 5% bovine serum albumin), again washed extensively with TBS/T, and incubated for 2 h with blocking solution containing HRP-conjugated rabbit-anti-mouse IgG, and signal was detected by ECL (Invitrogen, USA).

### 2.5. Quantitative Real-Time Reverse-Transcription Polymerase Chain Reaction

Total RNA was isolated from cell pellets with the RNeasy Mini Kit (Qiagen, Germany). Genomic DNA was removed from total RNA before cDNA synthesis with the RNase-free DNase set for DNase digestion during RNA purification (Qiagen). RNA was stored at −80°C. First-strand cDNA synthesis was performed for each RNA sample with the Sensiscript RT Kit (Qiagen). Random hexamers were used to prime cDNA synthesis.

mRNA expression was detected by real-time polymerase chain reaction (PCR) with the SYBR Green master mix (Applied Biosystems, USA). Thermocycler conditions comprised an initial holding at 50°C for 2 min and then at 95°C for 10 min. This was followed by a two-step PCR program consisting of 95°C for 15 sec and 60°C for 60 sec for 40 cycles. Data were collected and quantitatively analyzed on an ABI Prism 7900 sequence detection system (Applied Biosystems). The *β*-actin gene was used as an endogenous control to normalize for differences in the amount of total RNA in each sample. All quantities were expressed as number of folds relative to the expression of *β*-actin. Primers used in this study were as follows:

 
*β*-actin: sense 5′-GAGAAGAGCTACGAGCTGCCTGA-3′;
 antisense: 5′-ATCTTCATTGTGCTGGGTGCC-3′;
 PLK1: sense 5′-CTGCCTGCATCCCCATCTTC-3′;
 antisense: 5′-CACCATAGTGCGGGCGTAGC-3′;
 p53: sense 5′-TGACTGTACCACCATCCACTACAACTA-3′;
 antisense: 5′-GGCGGGAGGTAGACTGACCC-3′.

### 2.6. Immunohistochemistry

Immunohistochemistry was used to determine the expression of PLK1. Deparaffinized sections (4 *μ*m) were treated with 3% hydrogen peroxide in methanol for 10 min at RT to block endogenous peroxidase activity. Then the sections were incubated in citrate buffer for 10 min at 121°C and cooled to RT. After blocking with 10% bovine serum albumin for 1 h at RT, the slides were subsequently incubated overnight with anti-PLK1 at a dilution of 1 : 75. After extensive washing with PBS, the slides were incubated with secondary Ab for 30 min. The sections were then counterstained with DAB (Maxim, China).

A semiquantitative evaluation was performed by two independent observers, who were blinded to the clinical and pathological stage of the patients, on two separate occasions. The intensity of specimen staining was scored as follows: ++: strong; +: moderate; ±: weak; and −: not detectable. The extent of positive staining was further categorized into four groups (0, <30%, 30–60%, and >60%), on the basis of the proportion of the positively stained area [16].

### 2.7. Transmission Electronic Microscope

After human HCC cell line BCL-7402 was transfected with PLK1 siRNA for 48 h, the cells were washed and fixed with 2.5% glutaraldehyde in 0.1 M cacodylate buffer, pH 7.4 at 4°C. Samples were then rinsed in 0.1 M cacodylate buffer and postfixed for 1 h in 1% osmium tetroxide in the same buffer. This procedure was followed by dehydration and embedding in araldite resin. Ultrathin sections were double-stained with uranyl acetate-lead citrate and observed under a JEM-1200EX transmission electron microscope at 80 kV.

### 2.8. Statistical Analysis

The *χ* ^2^ and Fisher exact probability tests were used to examine associations between PLK1 expression and various other parameters including clinicopathological characteristics. Difference between two groups or among more than two groups was performed by Student's* t*-test and one-way ANOVA after analyzing the variance. The levels of significance were set to *α* ≤ 0.05.

## 3. Results

### 3.1. PLK1 Expression Is Significantly Upregulated in Human HCC

**Figure 1:**
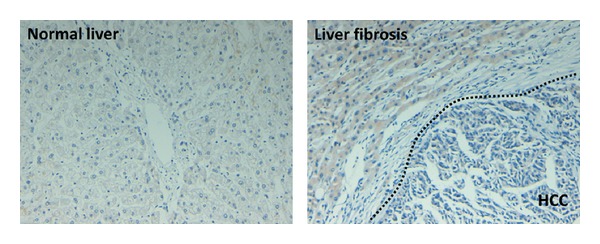
Expression of polo-like kinase 1 (PLK1) in hepatocellular carcinoma. Analysis of PLK1 expression in both normal liver and hepatocellular carcinoma (HCC) was performed by immunohistochemistry. Magnifications ×10 and magnification in insert ×40.

To investigate the role of PLK1 in human hepatocarcinogenesis, we determined its expression in normal human livers, HCCs, and surrounding cirrhosis tissues using immunohistochemistry. There was no PLK1 expression in normal liver (Figure 1). However, PLK1 expression in HCC samples was significantly increased compared with normal liver (Figure 1). To facilitate interpretation of the results, the patient was evaluated in different criteria. We found that PLK1 expression has no correlation with patient age, clinical stages, tumor nodules, tumor diameter, lymphatic metastasis, extrahepatic metastasis, with or without tumor thrombi in portal vein, HBV positive, HCV positive, and Child-Turcotte grading system for liver function evaluation. Interestingly, PLK1 expression was related to histological grading of tumor and capsule invasion. According to Sugihara grading criteria, HCCs were classified into well differentiated tumor, moderate differentiated tumor, and poor differentiated tumor. Notably, the expression of PLK1 in well differentiated tumor cells was significantly higher than moderate and poor tumor cells, but there was no significant difference between moderate tumor cells and poor tumor cells. In addition, PLK1 expression in different cells was different. This may be due to the different status of tumor cells. Therefore, we presume that PLK1 is associated with HCC development. Also, PLK1 expression in HCC without capsule invasion was more than that with capsule invasion (Table 1), which suggested less correlation of PLK1 expression with malignancy of HCC. More interesting is that, in 27 cases of cirrhosis from HCC, the expression of PLK1 in cirrhosis was positive either; moreover, 22 cases showed higher levels of PLK1 than that of cancer tissues (data not shown). We found that there is similar expression of PLK1 in cirrhosis with 3 paired HCC tissues and only 2 cases were lower than paired HCC tissues (data not shown). The difference of PLK1 expression in HCC nidus and surrounding cirrhosis tissues indicated that PLK1 may play an important role in HCC early development and its inhibition might contribute to the control of HCC.

### 3.2. PLK1 Inhibition Induces Growth Arrest in Hepatocellular Cell Lines

**Figure 2:**
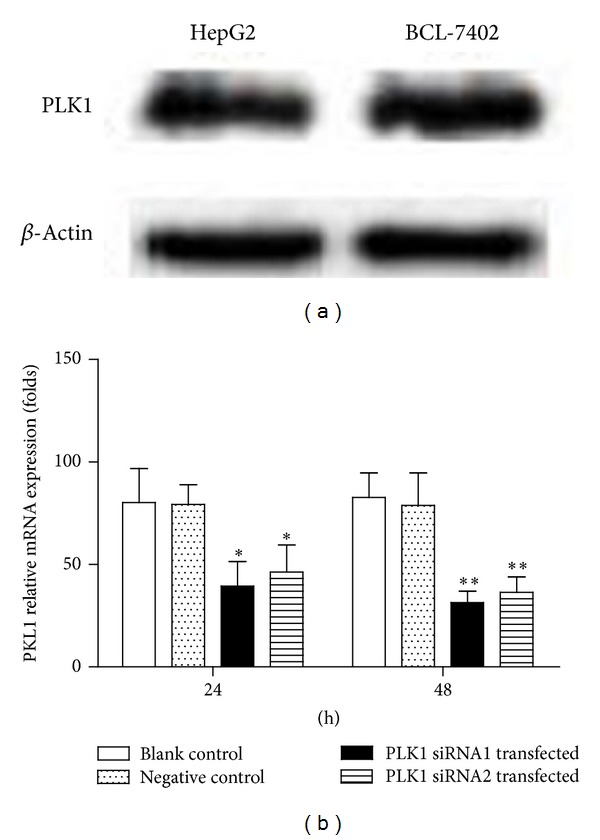
Polo-like kinase 1 (PLK1) expressions in hepatocellular carcinoma cell lines. (a) Western blot was used to determine the levels of PLK1 in HepG2 and BCL-7402 cell lines; beta-actin serves as the loading control. (b) Two designed siRNA for PLK1 were transfected to BCL-7402, respectively, and, 24 h and 48 h later, cells were collected. The expressions of PLK1 were examined by real-time PCR. Results are shown as mean ± SD. Data are representative of at least 3 independent experiments. *P < 0.05; **P < 0.01.

Using western blot, HepG2 and BCL-7402 cell lines were assayed for PLK1 expression. The assay on the above two cell lines revealed higher PLK1 expression (Figure 2(a)). In view of similar expression of PLK1 in both of the cell lines, we further select BCL-7402 cell lines for knockdown study. After interfering PLK1 expression with siRNA1 in BCL-7402, 24 h and 48 h later, we detected the PLK1 expression in PLK1 siRNA transfected BCL-7402, negative control, and blank control by real-time PCR. As shown in Figure 2(b), 24 h after siRNA1 transfection, PLK1 expression was decreased nearly 64% and 65%, which was compared with negative control and blank control, respectively, while, 48 h later, PLK1 expression decreased even more significantly, nearly 78% and 81%. Though PLK1 siRNA2 transfection in BCL-7402 also inhibited PLK1 expressions, the inhibitory efficiency is lower than that of PLK1 siRNA1 (Figure 2(b)). Thus, the role of PLK1 in HCC cell growth is investigated by assessing the consequence of PLK1 inactivation by siRNA1 in HCC cell lines.

**Figure 3:**
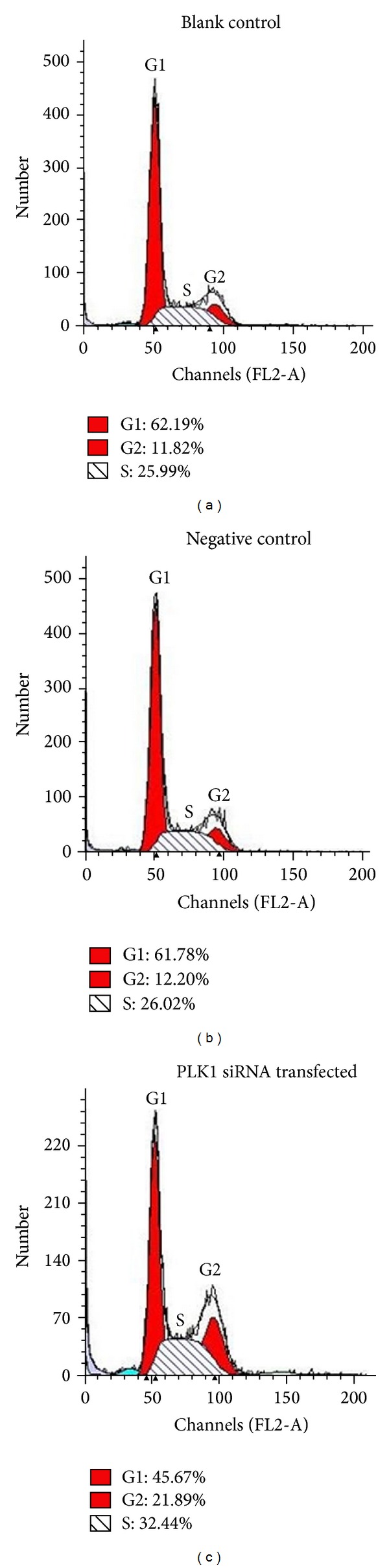
Knockdown of PLK1 in HCC promotes cell cycle arrest. BCL-7402 cells were transfected with PLK1 siRNA. 48 h later, cells were harvested and subjected to flow cytometry analysis for cell cycle progression. Data are representative of at least 3 independent experiments.

Since PLK1 is important during mitosis, we determined the effects of PLK1 siRNA on the cell cycle profile of BCL-7402 by flow cytometry. After 48 h PLK1 siRNA transfected BCL-7402, the proportion of cells in G2/M phase is lower than that of negative control and blank control; however, there was no significant difference between G0/G1 phase and M phase (Figure 3). All these results indicated that PLK1 knockdown prevents cells from undergoing mitosis. Therefore, we think PLK1 inhibition will be beneficial for HCC treatment.

### 3.3. PLK1 Downregulation Promotes Cell Apoptosis in Hepatocellular Cell Lines

**Figure 4:**
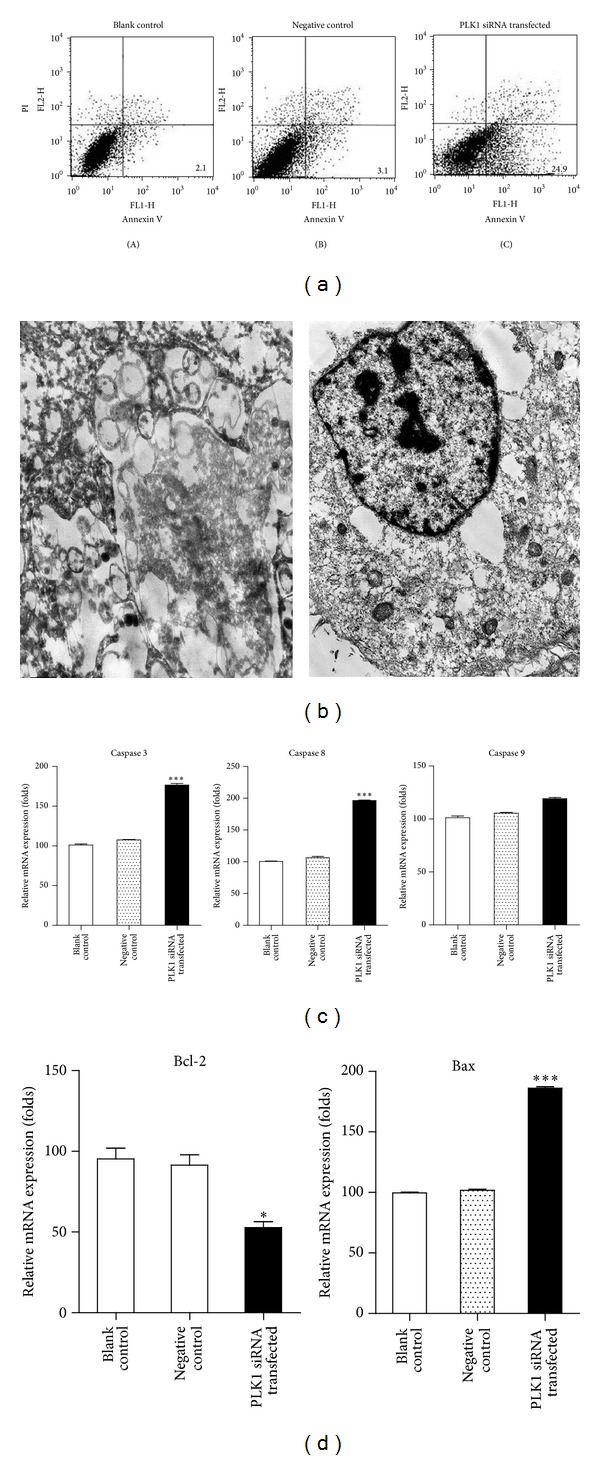
Knockdown of PLK1 in HCC accelerates cell apoptosis. BCL-7402 cells were transfected with PLK1 siRNA. 48 h later, (a) cells were harvested and subjected to annexin V and PI staining for apoptosis analysis by flow cytometry; (b) cells were observed using transmission electronic microscope (TEM); (c) cells were analyzed for caspase 3, caspase 8, and caspase 9 mRNA expression by real-time PCR; (d) cells were analyzed for Bcl-2 and Bax mRNA expression by real-time PCR. Results are shown as mean ± SD. Data are representative of at least 3 independent experiments. *P < 0.05; ***P < 0.001.

We then examined whether PLK1 has an important role in controlling cell apoptosis, for it can decide the tumor status. After knockdown of PLK1 in BCL-7402 for 48 h, cell apoptosis was assessed by annexin V and PI staining. Flow cytometry analysis showed that the apoptosis of PLK1 siRNA transfected BCL-7402 was significantly higher than that of negative control and blank control (Figure 4(a)). These results indicated that inhibition of PLK1 expression in tumor cells promotes cell apoptosis. Also, we performed the transmission electron microscopy to detect the morphology of PLK1 siRNA transfected BCL-7402. Consistent with the enhanced cell apoptosis in PLK1 siRNA transfected BCL-7402, these cells exhibited microvilli formation in cell membrane, chromatin condensation, karyorrhexis, and nuclear fragmentation, which happened in both early and late phases of apoptosis (Figure 4(b)). However, negative control and blank control hardly observed these apoptotic phenomena (data not shown). This result suggested that PLK1 knockdown in HCC cells can improve HCC development.

Based on the important role of caspase dependent signaling pathway in cell apoptosis, we further investigated caspase 3, caspase 8, and caspase 9 expression in PLK1 inhibited BCL-7402 by real-time PCR. Compared to control groups, PLK1 siRNA transfected BCL-7402 revealed significantly increased caspase 3 and caspase 8 expression, but not expression of caspase 9 (Figure 4(c)). In addition to the caspase signaling pathway, we also examined the balance of antiapoptotic molecular Bcl-2 and proapoptotic molecular Bax, which regulate cell apoptosis in mitochondria mediated signaling pathway. We found lower Bcl-2 expression and higher Bax expression in PLK1 siRNA transfected BCL-7402, which were compared to those of negative control and blank control (Figure 4(d)). Thus, the effect of PLK1 inhibition on cell apoptosis may be associated with mitochondria mediated signaling pathway.

### 3.4. p53 Is Involved in PLK1 Regulating Cell Cycle and Cell Apoptosis

**Figure 5:**
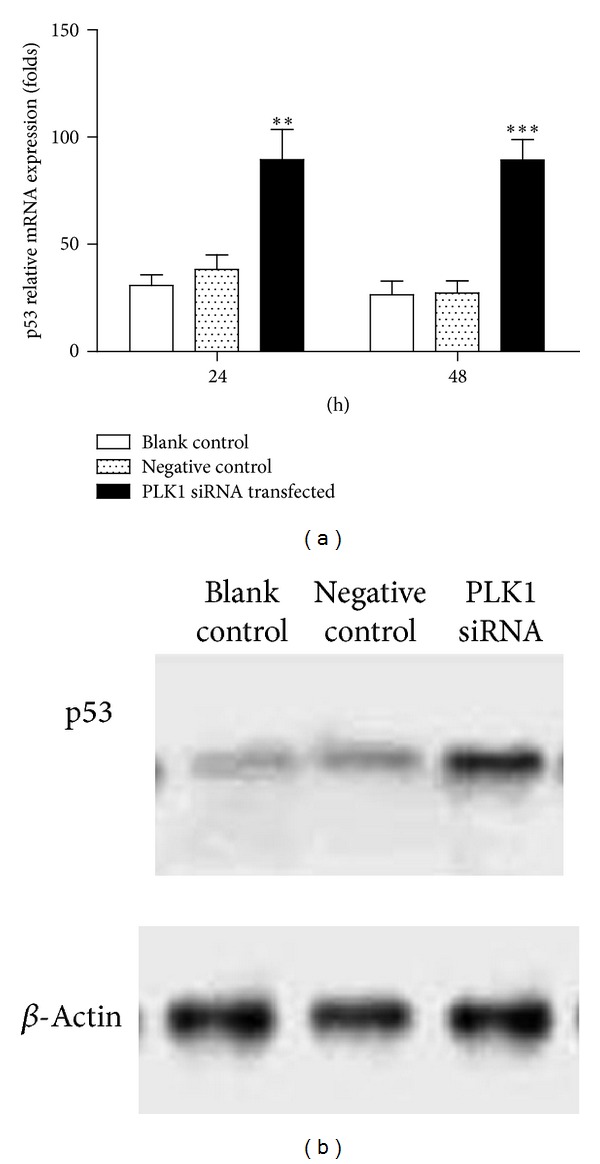
Knockdown of PLK1 in HCC upregulates the expression of p53. BCL-7402 cells were transfected with PLK1 siRNA. 48 h later, p53 expression was detected using real-time PCR (a) and western blot (b). Results are shown as mean ± SD. Data are representative of at least 3 independent experiments. **P < 0.01; ***P < 0.001.

Since p53 is important in the control of cell cycle progress and cell apoptosis, previous studies also have pointed that PLK1 can regulate the growth of cells in a p53-dependent manner in multiple types of carcinoma [17]. Therefore, we further detected the p53 expression in PLK1 siRNA transfected BCL-7402 by real-time PCR. We found that PLK1 inhibition promoted the expressions of p53 in both mRNA level and protein level (Figures 5(a) and 5(b)). Thus, it may be important evidence for p53 in complementing the regulatory role of PLK1 in controlling the growth of HCC cell lines.

## 4. Discussion 

Human HCC is one of the common and lethal tumors in the world. Though new strategies are available, the survival of unresectable HCC patients is still poor. Thus, new molecules for diagnosis and therapeutic targets should be identified and targeted to improve HCC patients outcome. In this study, we found upregulated expression of PLK1 in human HCCs. Meanwhile, our data further indicated that PLK1 knockdown inhibited the growth of HCC cell lines and promoted the apoptosis of HCC cell lines. All these effects were associated with p53 signaling pathway. Therefore, we presume that PLK1, as the potential target, will be beneficial for the diagnosis and treatment of HCC.

In consistence with previous studies, we demonstrated that PLK1 upregulation in the liver was found in HCC patients [18, 19]. After evaluating by different clinicopathological variables, age, tumor size, tumor nodule, clinical stage, HBV positive, HCV positive, liver function, and with or without tumor thrombi in portal vein showed less correlation with PLK1 expression. However, the higher expression of PLK1 in HCC was found in well differentiated tumor cells and tumor without capsule invasion, but not in moderate and poor differentiated cells and tumor with capsule invasion. Moreover, though PLK1 expression was correlated with metastasis in many types of carcinoma, there was less relationship between PLK1 expression and HCC metastasis, including lymphatic metastasis and extrahepatic metastasis [14, 18]. Therefore, contrary to the positive relationship of PLK1 and some types of tumor malignance, less correlation of PLK1 and HCC malignancy was observed in the present study. This may be due to the special tissue microenvironment where tumor happened. Meanwhile, we found that, even in the same HCC sample, different levels of PLK1 distributions in different subgroup of tumor cells were detected. This finding indicated that PLK1 may play a different role in regulating HCC stem cells to differentiate into different subtypes of tumor. Thus, in controlling well differentiated HCC cells development, PLK1 may be important, while, in moderate or poor differentiated HCC cells, PLK1 maybe need to collaborate with other factors. Also, given that the distinct sites of phosphorylation in PLK1 will influence different stage of cell growth [19, 20], further studies are still needed to investigate whether the different sites of phosphorylation in PLK1 will affect the differentiation of HCC stem cells. In addition, the similar elevated levels of PLK1 expression in HCCs and surrounding cirrhosis suggested that PLK1 can serve as the prognostic standard to judge the possibility of canceration of cirrhosis tissue. Thus, PLK1 may be an important diagnostic marker to evaluate HCC development.

Furthermore, we explored the effect of PLK1 on cell cycle modulation in human HCC cell lines. Consistent with previous studies, our data demonstrated the regulatory role of PLK1 in both G2/M phase of the cell cycle and the apoptotic process [21, 22]. In the present study, through inhibiting Bcl-2 expression, promoting Bax expression and PLK1 downregulation promoted cell apoptosis. It has been demonstrated that the knockdown of PLK1 leads to an increased expression of Bax in Hela cells, which contribute to the apoptosis of cells [23]. Bax is a key component for cellular induced apoptosis through mitochondrial stress. With apoptotic stimulation, oligomers of Bax interact with mitochondrial membrane, which at last initiates the caspase activation pathway for apoptosis [24]. We also detected the enhanced levels of caspase 3 and caspase 8 in PLK1 knockdown cells. Therefore, we put forward a hypothesis that PLK1 might be a crucial therapeutic target in HCC, due to the activation of proapoptotic pathway. We found that PLK1 inhibition can promote p53 expression, which in turn can contribute to cell cycle arrest and cell apoptosis. Recently, some new findings have pointed that PLK1 is able to inhibit apoptosis in a p53-dependent manner in a variety of carcinomas [8, 25]. PLK1 can interact with the DNA binding domain of p53, thereby decreasing its stability and transcriptional activity [17]. Thus, p53 is a major target for PLK1 controlling the growth of carcinoma cells. Besides p53, many researchers have found that p73 is another target of PLK1 and its expression was elevated in PLK1 silenced HCC cell lines with or without p53 expression [26]. The enhancement of p73 was also observed in MCF7 breast cancers expressing the p53; however, p53 is independent of the process of p73 induction by PLK1 [27]. Though some targets have been verified, we cannot exclude other molecules involved in PLK1 regulating the growth of HCC cell lines, such as Orc2, centrobin/NIP2, and HsCYK-4. All of them have been indicated to be targeted by PLK1, their activation following by promoting microtubule stabilization or regulating the onset of cell division. Therefore, it is still necessary to investigate whether these molecules were involved in PLK1 pathway modulating the growth of HCC.

## 5. Conclusions

Taken together, in the present study, we have demonstrated that upregulation of PLK1 expression may be an early diagnostic marker for the canceration of cirrhosis and the development of HCC, but less correlation between PLK1 and metastasis was observed. Inhibiting PLK1 expression in HCC cell line can significantly decrease cell proliferation and increase cell apoptosis. In addition, we found that these processes may be involved in p53 mediated signaling pathway. Our study provides new evidence for the involvement of PLK1 in HCC development process and puts forward the possibility of PLK1 serving as a target for HCC diagnosis and treatment.

## Figures and Tables

**Table 1:** The relationship of PLK1 expression and HCC clinicopathological variables.

Clinicopathological factors	*n*	PLK1 expression			Positive incidence
		None	Low	High	
Age (yr)					
>50	39	8	12	19	78.5%
≤50	28	8	9	11	71.4%
Clinical stage					
Phases I + II	46	12	14	20	73.9%
Phase III	21	4	7	10	81.0%
Tumor nodule					
1	48	12	12	24	75.0%
≥2	19	4	9	6	78.9%
Tumor diameter					
>5 cm	44	12	14	18	72.7%
≤5 cm	23	4	7	12	82.6%
Lymphatic metastasis					
Yes	5	2	2	1	60.0%
No	62	14	19	29	77.4%
Extrahepatic metastasis					
Yes	10	4	3	3	60.0%
No	57	12	18	27	78.9%
Capsule invasion					
Yes	27	10	8	9	63.0%**
No	40	6	13	21	85.0%
Tumor thrombi in portal vein					
Yes	15	4	5	6	73.3%
No	52	12	16	24	76.9%
Histological differentiation (Sugihara grading criteria)					
Well-	26	1	7	18	96.2%*
Moderate/poor-	41	15	14	12	63.4%
Total	**67**	**16**	**21**	**30**	**76.1%**

The *P* value is less than 0.01, when HCC with capsule invasion is compared with HCC without capsule invasion. The *P* value is less than 0.05, when HCC in well differentiation is compared with HCC in moderate or poor differentiation.

*P < 0.05, **P < 0.01.
